# Rapid health technology assessment of galantamine for the treatment of Alzheimer’s disease: A review

**DOI:** 10.1097/MD.0000000000042744

**Published:** 2025-06-06

**Authors:** Bingke Xing, Jingran Yang, Hui Hua, Rong Jiang

**Affiliations:** a School of International Pharmaceutical Business, China Pharmaceutical University, Nanjing, Jiangsu, China; b The Research Center of National Drug Policy and Ecosystem, Nanjing, Jiangsu, China.

**Keywords:** Alzheimer disease, cost-effectiveness, efficacy, galantamine, rapid health technology assessment, safety

## Abstract

This study evaluates the efficacy, safety, and economics of galantamine in the treatment of Alzheimer disease (AD) patients, and to provide evidence-based reference for optimizing drug selection, dosing strategies, and reimbursement policies in clinical practice. We used galantamine, systematic review, cost, and their synonym as keywords. The PubMed, Web of Science, Cochrane Library, China National Knowledge Infrastructure, Weipu, Wanfang database, and Health Technology Assessment organization websites were searched to retrieve studies regarding galantamine for the treatment of AD from database creation to September 20, 2023. We extracted information and pooled the results of the studies for qualitative analyses instead of quantitative meta-analysis. This study incorporated 39 reports. Regarding effectiveness, galantamine significantly improved cognitive function, living capacity, mental behavior, and global functioning of the AD patients compared with the placebo. However, based on available data, it was not possible to infer whether galantamine was more effective than the other acetylcholinesterase inhibitor drugs. AD patients treated with galantamine showed a higher incidence of adverse gastrointestinal effects compared to those treated with placebo. Galantamine showed a higher incidence of adverse gastrointestinal effects than donepezil but a lower rate than rivastigmine. Regarding economics, compared with the placebo and non-pharmacological treatments, galantamine prolonged the duration of full-time care and in some cases even reduced the overall cost of galantamine use. Galantamine showed good efficacy, cost-effectiveness, and mild adverse effects in the treatment of patients with AD by prolonging the duration of full-time care and saving the cost compared with the placebo and non-pharmacological.

## 1. Introduction

Alzheimer disease (AD) is a chronic progressive neurodegenerative disease with no cure. It is characterized by progressive memory loss, cognitive and language dysfunction, hallucinations, and neurological symptoms. As the disease progresses, AD patients are associated with several comorbidities and gradually lose their ability to care for themselves and become totally dependent on others for their daily living needs. Therefore, AD is associated with significant economic and public health burden. The current data shows that approximately 47.5 million people are estimated to be living with AD worldwide; the number of AD patients are projected to reach 82 million in 2030 and exceed 152 million in 2050.^[1]^ The annual cost of treating patients is increasing every year. In China, the total annual costs for treating AD patients were estimated to be $167.74 billion in 2015, and are expected to reach $1887.18 billion by 2050.^[2]^

Given the substantial impact of AD, it is crucial to explore the current treatment options and their effectiveness. The pathogenesis of AD is complex. Studies have indicated that the loss of cholinergic neurons plays a critical role in the disease, affecting cognitive function.^[3]^ Another key contributor is the toxicity caused by amyloid-beta (Aβ) proteins, which can damage brain cells. The formation of neurofibrillary tangles, resulting from abnormal tau protein, further disrupts the internal structure of neurons. Additionally, oxidative stress and excitotoxicity contribute to neuronal damage, while a decline in estrogen levels may also exacerbate the disease in some individuals. Although currently there are no specific drugs or curative methods for AD,^[4]^ the cognitive function can be improved and the disease progression can be delayed through medications, non-pharmacological treatments and daily care. Acetylcholinesterase inhibitors (AChEIs) are currently the first-line drugs for the treatment of mild to moderate AD. Acetylcholine is mediator that helps transmit signals between nerve cells. Acetylcholinesterase (AChE) can rapidly break down acetylcholine and convert it into inactive components to block neurotransmission. AChEIs inhibit AChE’s activity by binding to it. The long-term presence of acetylcholine allows connections between nerve cells to be maintained and enhances neurotransmission, improving cognitive function and memory in patients with AD. Galantamine, donepezil, and carboplatin are all included in medication guidelines such as the Chinese Guideline for the Diagnosis and Treatment of Alzheimer Disease Dementia,^[5]^ Chinese Expert Consensus on the Diagnosis and Treatment of Mild Cognitive Impairment due to Alzheimer Disease 2021,^[6]^ Practice Guideline for the Treatment of Patients With Alzheimer Disease and Other Dementias.^[7]^ One of the most promising AChEIs is galantamine, a second-generation inhibitor with multiple mechanisms of action. Galantamine can increase the concentration of acetylcholine in the brain by inhibiting cholinesterase and modulating nicotinic receptors outside the brain. At the same time, it can also block the Ca^2+^, activated K^+^ channels to promote the release of neurotransmitters and intervene in the expression and metabolism of amyloid precursor proteins, which can in turn alleviate the clinical symptoms of patients with AD.^[8]^ Galantamine was first marketed as a cholinesterase inhibitor in Austria in 1996, and was subsequently approved in the European Union as a treatment for various degrees of AD.^[9]^ On February 28, 2001, galantamine was approved by the US FDA for the treatment of mild to moderate AD, and was approved for marketing in China in 2003, and has been incorporated into the China’s National Reimbursement Drug List.

To fully understand the role of galantamine in AD treatment, it is essential to evaluate its clinical value through comprehensive studies. Health technology assessment (HTA) utilizes methods of evidence-based medicine to systematically evaluate the technical characteristics, effectiveness, safety, and economics of health technologies, including pharmaceuticals, and provides decision makers with relevant scientific information to support formulation of health policy. Rapid HTA simplifies the systematic review (SR) methodology for specific issues to summarize and analyze data in a short period of time. Galantamine is one of the first-line drugs for the treatment of AD patients, and existing studies have assessed the clinical value of galantamine mainly through randomized controlled trials, meta-analysis, and cost-effectiveness analysis. A large amount of data has been accumulated around the clinical efficacy, safety, and economics of galantamine, but there is a lack of comprehensive assessment of its clinical benefits. The aim of this study was to conduct a rapid HTA assessment of the safety, efficacy, and economics of galantamine for the treatment of AD using the best available evidence to provide the most accurate evidence for healthcare decision-making.

## 2. Material and methods

### 2.1. Literature search strategies

We searched the PubMed, Cochrane Library, Web of science, China National Knowledge Infrastructure, Weipu, Wanfang databases, and the official websites of HTA organizations at home and abroad such as the National Institute for Health and Care Excellence (https://www.nice.org.uk/) and the Institut fur Qualitat und Wirtschaftlichkeit im Gesundheitswesen (https://www.iqwig.de/en/) from the time of database construction to September 20, 2023. The following search terms were used: (dementia OR Alzheimer disease) AND (Galanthamine OR Galantamine OR Nivalin OR Reminyl) AND (systematic review OR meta-analysis OR economics OR cost). The search strategy is shown in Table S1, Supplemental Digital Content, https://links.lww.com/MD/P121.

### 2.2. Inclusion and exclusion criteria

The inclusion criteria were as follows: (1) the article was a HTA report, SR or meta-analysis, and economic study, with the language of articles limited to Chinese and English; (2) the study population was AD patients; (3) the studies compared galantamine with other therapeutic drugs, placebo, or no drug; and (4) the specific outcome indicators included were are shown below.

- The efficacy indicators: Mini-Mental State Examination (MMSE) score, Alzheimer Disease Assessment Scale-Cognitive subscale (ADAS-cog) score, Activity of Daily Living (ADL) score, Alzheimer Disease Cooperative Study-Activities of Daily Living (ADCS-ADL) score, Disability Assessment for Dementia (DAD) score, Neuropsychiatric Inventory (NPI) score, Clinical Global Impression of Change (CGIC) score, and Clinician’s Interview-Based Impression of Change Plus caregiver input (CIBIC+) score.
- The safety indicators: the incidence of adverse events and the dropout rates.
- Economic indicators: the full-time care (FTC) and cost, Quality Life-adjusted Years (QALYs), and Incremental Cost-Effectiveness Ratios.

The exclusion criteria were as follows: (1) study design included animal experiments and basic experimental studies; (2) duplicate studies; (3) literature with unavailable data.

### 2.3. Literature screening and data extraction

Literature screening, data extraction, and cross-checking were conducted independently by 2 researchers (Bingke Xing and Jingran Yang). The disagreements were resolved after discussion with a third researcher (Rong Jiang). The data extraction included authors, year of publication, number of included studies, sample size, interventions, controls, and outcome indicators.

### 2.4. Literature quality assessment

Two researchers (Bingke Xing and Jingran Yang) independently assessed the quality of included studies to fully analyze the quality level of the existing literature, identify gaps in the current research, and provide recommendations for future research. The quality of SR/meta-analysis was assessed using the AMSTAR rating system, which consisted of 16 entries.^[10]^ Among them, entries 2, 4, 7, 9, 11, 13, and 15 are key entries, and the evaluation result of each entry is categorized as Yes, Partial Yes, or No. The evaluation result of No is considered non-compliant. Zero or 1 non-compliant non-critical entries are rated as high quality. One or more non-conforming non-key entries are rated as moderate quality. One non-conformity of key entries with or without non-conformity of non-key entries is rated as low quality. One or more non-conforming key entries with or without non-conforming non-key entries are rated as very low quality. The Consolidated Health Economic Evaluation Reporting Standards (CHEERS) scale was used to evaluate the economic research studies.^[11]^ HTA checklist developed by the International Association of Health Technology Assessment Organizations was used to assess the quality of reports with HTA.^[12]^

### 2.5. Evidence synthesis and analysis

As the included study types were highly heterogenous, this study used descriptive methods to assess outcomes. The pooled findings of the included HTA reports, SR/meta-analyses, and economics reports were subjected to qualitative descriptive analyses according to the study design, patient population, and intervention/control measures of each stud by using Microsoft Excel 2019.

## 3. Results

### 3.1. Literature search results

**Figure 1.**
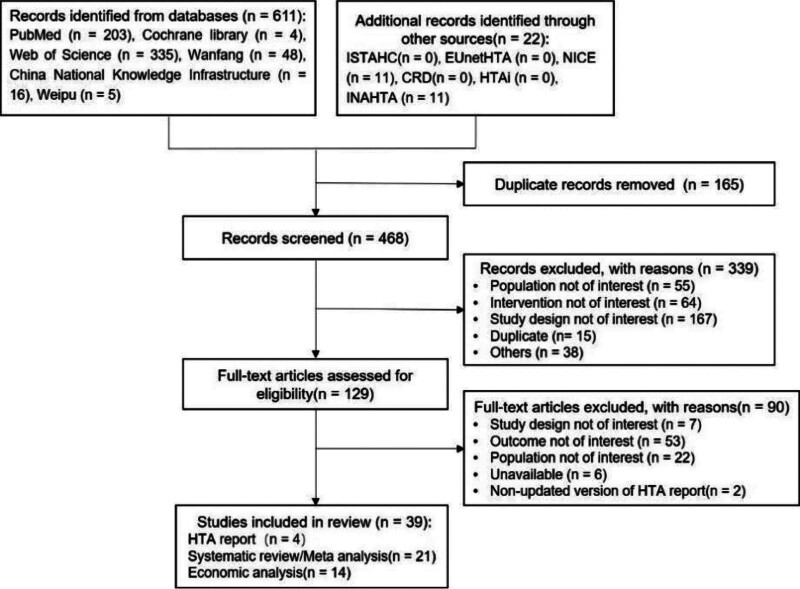
Flow chart of study screening.

Figure 1 shows the study selection protocol used in this study. Firstly, we obtained 633 reports after searching various online databases. Then, we eliminated duplicate reports using the EndNote software and retained 468 reports. The titles and abstracts of these reports were initially screened and 339 reports were excluded. After re-screening the full text of the remaining 129 reports, we included 39 relevant reports, including 4 HTA reports, 21 SR/meta-analyses, and 14 economics reports for the final analyses. Figure 1 shows the study screening process.

### 3.2. Main characteristics and quality evaluation of the included literature

The basic characteristics and the quality evaluation results of the included studies are shown in Tables 1–3.^[13–51]^ The number of studies included in each SR/meta-analysis ranged from 5 to 142 and the sample sizes ranged from 802 to 33,889 AD subjects. The time-frame for the pharmacoeconomic studies ranged from 6 months to 10.5 years and included countries such as Canada, Sweden, Netherlands, and so on. Three out of 4 HTA reports were obtained from National Institute for Health and Care Excellence, whereas the remaining HTA report was obtained from Institut fur Qualitat und Wirtschaftlichkeit im Gesundheitswesen. Among these, all the 4 HTA reports evaluated the clinical effectiveness of galantamine over placebo and 2 HTA reports evaluated the cost-effectiveness of galantamine.

**Table 1:** Basic characteristics and quality evaluation of the included SR/meta-analysis.

Study	Number of included studies	Study population	Total cases	Comparison group	Control group	Outcome index	Literature quality rating
Lanctôt et al (2003)^[13]^	16	AD	7954	Donepezil 1, 3, 5, 10 mg/d, rivastigmine 1–4, 6–12 mg/d, galantamine 8, 16, 24, 32, 36 mg/d	Placebo	Withdrawal rate, incidence of adverse effects	Low
Kurz and Van Baelen (2004)^[14]^	54	AD	/	Ginkgo biloba 80–600 mg/d, donepezil 5, 10 mg/d, rivastigmine 6–12 mg/d, galantamine 16, 24 mg/d	Placebo	ADAS-cog, withdrawal rate	Very low
Harry et al (2005)^[15]^	8	Mild to moderate AD	3352	Donepezil 5, 10 mg/d, galantamine 8, 16, 24, 32, 36 mg/d	Placebo	ADAS-cog	Very low
Takeda et al (2006)^[16]^	26	Mild to moderate AD	/	Donepezil 5, 10 mg/d, galantamine 8–36 mg/d, rivastigmine flexible dose	Placebo	ADAS-cog, MMSE, incidence of adverse effects	Very low
Loy and Schneider (2006)^[17]^	10	Mild to moderate AD	6805	Any oral dose of galantamine	Placebo	ADAS-cog, ADCS-ADL, CIBIC-plus or CGIC, DAD, NPI, withdrawal rate, incidence of adverse effects	Low
Hansen et al 2008^[18]^	33	Mild to moderate AD	/	Donepezil 5–10 mg/d, rivastigmine 6–12 mg/d, galantamine 8–32 mg/d	Placebo	ADAS-cog, ADCS-ADL, DAD, CIBIC-plus, NPI, withdrawal rate	Low
Campbell et al (2008)^[19]^	12	Mild to moderate AD	4921	Donepezil 10 mg/d, rivastigmine capsule 3–12 mg/d or a skin patch 20 cm^2^/d, galantamine 16–24 mg/d	Placebo	NPI	Very low
Rodda et al (2009)^[20]^	14	AD	6110	Donepezil 5–10 mg/d, rivastigmine 12 mg/d or a skin patch 9.5, 17.4 cm^2^/d, galantamine 8, 16–32 mg/d	Placebo	NPI	Very low
Lockhart et al (2009)^[21]^	12	Mild to moderate AD	/	Donepezil	Rivastigmine, galantamine	Incidence of adverse effects	Low
Kavanagh et al (2011)^[22]^	18	Mild to moderate AD	/	Galantamine 16–24 mg/d	Placebo	ADAS-cog	Very low
Xiaojuan (2011)^[23]^	5	Mild to moderate AD	802	Galantamine	Placebo	ADAS-cog, MMSE, incidence of adverse effects	Very low
Sisi et al (2014)^[24]^	11	AD	/	Galantamine 4–36 mg/d	Placebo	ADAS-cog, ADL, CIBIC-plus, DAD, MMSE, NPI, incidence of adverse effects	Low
Jiang et al (2015)^[25]^	11	AD	4074	Galantamine 16–40 mg/d	Placebo	ADAS-cog, ADL, CIBIC-plus, MMSE, NPI, withdrawal rate, incidence of adverse effects	Low
Kobayashi et al (2016)^[26]^	21	Mild to moderate AD	9509	Donepezil 5, 10 mg/d, rivastigmine 2–12 mg/d or a skin patch 18 mg/d, galantamine 16, 18, 24 mg/d	Placebo	ADAS-Cog, CIBIC-plus, CGIC, NPI, incidence of adverse effects	Very low
Qian et al (2017)^[27]^	30	Mild to moderate AD	–	Donepezil 5, 10 mg/d, galantamine 16–24 mg/d, 32 mg/d, huperzine A 400 μg/d, rivastigmine 1–12 mg/d	Placebo	ADAS-Cog	Low
Tricco et al (2018)^[28]^	142	AD	33,889	Donepezil, galantamine, rivastigmine, memantine	No treatment, placebo, best supportive care	ADAS-Cog, CIBIC-plus, incidence of adverse effects	High
Dou et al (2018)^[29]^	41	AD	18,898	Donepezil 5, 10 mg/d, galantamine 24, 32 mg/d, rivastigmine 12 mg/d or a skin patch 5, 10, 15 cm^2^/d, memantine 20 mg/d	Placebo or other treatments	ADAS-cog, ADL, incidence of adverse effects	Low
Liang et al (2018)^[30]^	35	AD	9820	Donepezil 10 mg/d, rivastigmine 24 mg/d, galantamine 18 mg/d, memantine 20 mg/d, huperzine A 0.2 mg/d, Tacrine 80 mg/d	Cognitive drugs, or in any combination (no treatment, placebo, best supportive care)	MMSE	Very low
Thancharoen et al (2019)^[31]^	27	Mild to moderate AD	9774	Ginkgo biloba 240 mg/d, donepezil 5, 10 mg/d, galantamine 16–24 mg/d, rivastigmine 6–12 mg/d or a skin patch 9.5 mg/24 h, memantine 20 mg/d	Placebo	ADAS-cog, ADCS-ADL, DAD, NPI, CIBIC-plus, ADCS-CGIC, CGIC, withdrawal rate, incidence of adverse effects	Low
Li et al (2019)^[32]^	36	Mild, moderate, severe AD	–	Donepezil 5, 10 mg/d, rivastigmine 6–12 mg/d, galantamine 16, 18, 24, 32, 36 mg/d, memantine 20 mg/d	Placebo	ADAS-cog, ADCS-ADL, NPI, CIBIC-plus, withdrawal rate, incidence of adverse effects	Very low
Zhang et al (2020)^[33]^	37	Mild to moderate AD	14,705	Ginkgo biloba 160, 240 mg/d, donepezil 5, 10 mg/d, galantamine 24, 32 mg/d, rivastigmine 12 mg/d or a skin patch 5, 10, 15 cm^2^/d, memantine 20 mg/d, huperzine A 200, 400 µg/d/.	Placebo	ADAS-cog, ADCS-ADL, NPI, MMSE, withdrawal rate, incidence of adverse effects	Very low

ADAS-cog = Alzheimer Disease Assessment Scale-cog, ADCS-ADL = Alzheimer Disease Cooperative Study-ADL, ADCS-CGIC = Alzheimer Disease Cooperative Study-Clinical Global Impression of Change, ADL = Activity of Daily Living, CGIC = Clinical Global Impression of Change, CIBIC-plus = Clinician’s Interview-Based Impression of Change Plus caregiver input, DAD = Disability Assessment For Dementia, MMSE = Mini-Mental State Examination, NPI = Neuropsychiatric Inventory, – = not reported in the literature.

The overall quality of the 21 SR/meta-analyses were low. Only 1 SR/meta-analysis achieved high quality assessment rating. Majority of the SR/meta-analyses reports did not include the pre-design program (entry 2). The specific evaluation results are shown in Table S2, Supplemental Digital Content, https://links.lww.com/MD/P122. The quality of the 14 economics studies included in this study were evaluated using the CHEERS 2022 scale. The average percentage of the 14 articles meeting the CHEERS 2022 standard was 77.6%. Therefore, the overall quality of the included pharmacoeconomic studies was average. The specific evaluation results are shown in Table S3, Supplemental Digital Content, https://links.lww.com/MD/P124. The HTA checklist included 14 evaluation criteria. The overall HTA report quality rating was good. The results of the HTA evaluation are shown in Table S4, Supplemental Digital Content, https://links.lww.com/MD/P125.

### 3.3. Efficacy evaluation of galantamine treatment in AD patients

#### 3.3.1. Effects of galantamine treatment on the cognitive function of AD patients

We evaluated the effects of galantamine on cognitive function of AD patients by comparing the MMSE and ADAS-cog scores in different intervention groups. Four HTA reports showed that galantamine significantly improved cognitive function compared to the placebo.^[48–51]^ Two HTA reports also showed that the benefits of galantamine treatment increased over time. AD patients treated with galantamine showed significantly higher improvement in cognitive function at 21 to 26 weeks (WMD = -2.96, 95% CI [-3.41, 2.51], *P* < .001) compared to 12 to 16 weeks (WMD = -2.39, 95% CI [-2.80, -1.97], *P* < .001).^[50,51]^

Seventeen SR/meta-analyses reported changes in the MMSE and ADAS-cog scores after galantamine treatment.^[14–18,22–33]^ In all the included studies, the MMSE and ADAS-cog scores in the galantamine group were significantly higher than those in the placebo group. Takeda et al compared the efficacies of donepezil with galantamine and reported that the mean change in the MMSE scores was significantly higher in the donepezil group (1.6 points) compared with the galantamine group (0.8 points) (*P* < .05).^[16]^ Furthermore, Takeda et al and Thancharoen et al reported that the improvement in ADAS-cog scores was lower in the galantamine group compared with the donepezil group.^[16,31]^ Moreover, Kobayashi et al, Dou et al, and Zhang et al reported that galantamine was the most effective treatment by comparing the improvements in ADAS-cog scores after treatment with galantamine, rivastigmine, donepezil, and memantine.^[26,29,33]^

#### 3.3.2. Effects of galantamine treatment on the daily living capacity of AD patients

We evaluated the effects of galantamine on the daily living capacity of AD patients by comparing the ADL, ADCS-ADL, and DAD scores in different intervention groups. Four HTA reports showed that galantamine treatment significantly improved the living capacity of the AD patients compared to the placebo group.^[48–51]^ The pooled estimates of ADCS-ADL and DAD scores of patients with AD showed significant functional benefit from galantamine compared with the placebo (SMD = 0.27, 95% CI [0.18, 0.34], *P* < .001).^[50]^

We included 8 SR/meta-analyses for evaluating the effects of galantamine on the daily living capacity of AD patients. Three SR/meta-analyses reported changes in the ADL scores. Among these, 2 SR/meta-analyses reported that the ADL scores did not significantly change in the galantamine group compared to the placebo group.^[24,25]^ Dou et al demonstrated that treatment with 24 mg/day galantamine significantly improved the ADL scores compared to the placebo group.^[29]^ Differences in ADL score results may be related to heterogeneity among the included studies. Heterogeneity implies that there were differences between the included studies in terms of AD patient characteristics, galantamine dosage, and duration of treatment, which may have led to different results and made it difficult to accurately reflect the efficacy of galantamine. Three SR/meta-analyses reported changes in the ADCS-ADL scores. Loy et al reported that ADCS-ADL scores decreased slightly in AD patients treated with 16 mg/d and 24 mg/d galantamine compared to the placebo group.^[17]^ Li et al and Zhang et al reported that galantamine treatment increased the ADCS-ADL scores compared to the placebo group.^[32,33]^ Two SR/Meta-analyses measured the effects of galantamine treatment on the living capacity by pooling the ADCS-ADL and DAD scores. Hansen et al reported that 16 to 32 mg/d galantamine treatment significantly improved the daily living capacity compared to the placebo group (SMD = 0.27, 95% CI [0.18, 0.36]).^[18]^ Thancharoen et al showed that galantamine was significantly better than memantine in delaying the functional impairment in the AD patients (SMD = 0.28, 95% CI [0.06, 0.50]).^[31]^

#### 3.3.3. Effects of galantamine treatment on the psycho-behavioral improvement in AD patients

We evaluated the effects of galantamine on the psychiatric behavior of the AD patients by comparing the NPI scale scores between different intervention groups. Three HTA reports demonstrated that treatment with galantamine significantly improved the psychiatric behavior of AD patients compared with the placebo group when analyzed at weeks 21 to 26 (WMD = -1.46, 95% CI [-2.59, -0.34], *P* = .012).^[49–51]^

Ten SR/meta-analyses were included to analyze the effects of galantamine on the psychiatric behavior of AD patients. Two SR/meta-analyses reported that treatment with galantamine, donepezil, or rivastigmine did not significantly improve the NPI scores of patients with AD.^[26,33]^ The remaining 8 SR/meta-analyses reported that galantamine treatment significantly improved the NPI scores compared to the placebo.^[17–20,24,26,31,32]^ Thancharoen et al reported that galantamine treatment significantly improved the behavioral symptoms compared with the placebo and treatment with donepezil and rivastigmine (SMD = -0.15, 95% CI [-0.26, -0.04]), as well as memantine (SMD = -0.25, 95% CI [-0.48, -0.03]).^[31]^

#### 3.3.4. Effects of galantamine treatment on the global functional improvement in AD patients

We compared the ADCS-CGIC and CIBIC-plus scores of different intervention groups to determine the effects of galantamine on the global functions of patients with AD. Four HTA reports demonstrated that galantamine significantly improved the global function of patients with AD and the CIBIC-plus scores of the galantamine group were significantly higher than the placebo group when followed-up at week 26 (WMD = -0.20, 95% CI [-0.30, -0.09], *P* < .001).^[48–51]^

Subsequently, we included 8 SR/meta-analyses to evaluate the effects of galantamine on the global function of the AD patients. Five SR/meta-analyses reported that galantamine significantly improved the CIBIC-plus scores in patients with AD.^[18,24,25,28,42]^ Tricco et al compared the efficacies of AChEIs based on the improvements in the CIBIC-plus scores and reported that the beneficial effects of galantamine were superior to donepezil (MD = -3.47, 95% CI [-6.66, -0.26]), oral rivastigmine (MD = -3.41, 95% CI [-6.62, -0.21]), and transdermal rivastigmine (MD = -3.59, 95% CI [-6.78, -0.39]).^[28]^ Two SR/meta-analyses reported changes in the CIBIC-plus and CGIC scores after treatment of AD patients with galantamine. Loy et al reported that the proportion of AD subjects with improved or unchanged CIBIC-plus and CGIC scores increased significantly at galantamine dose levels > 8 mg/day.^[17]^ Kobayashi et al reported that the CIBIC-plus and CGIC scores of AD patients treated with donepezil and rivastigmine were significantly higher than those treated with galantamine.^[26]^ Thancharoen et al performed a pooled analysis of the CIBIC-plus, ADCS-CGIC, and CGIC scores in patients with AD and reported that the proportion of AD patients with improved or unchanged global function from baseline were significantly higher in the galantamine group compared to the placebo group (RR = 1.20, 95% CI [1.01, 1.44]); moreover, donepezil showed more effective than galantamine in improving the clinical global functioning of patients with AD (RR = 1.40, 95% CI [1.09, 1.80]).^[31]^

### 3.4. Safety evaluation of galantamine treatment in AD patients

According to 4 HTA reports, gastrointestinal side effects were the most common adverse effects in the AD patients.^[48–51]^ Furthermore, a rapid increase in the dose of galantamine by 8 mg per week caused nausea in one-third of the treated patients and vomiting in one-fifth of the treated patients. However, there were no significant differences in the serious adverse effects between the galantamine and placebo groups (OR = 1.08, 95% CI [0.84, 1.40]).^[49]^

Thirteen SR/meta-analyses reported adverse effects in patients with AD after galantamine treatment. Among these, the results of 10 studies showed that compared with the placebo group, the incidence rates of adverse effects were higher in the galantamine group, including gastrointestinal side effects such as nausea, vomiting, and loss of appetite, and neurological side effects such as dizziness and headache.^[13,19–22,24,25,27–29]^ Three studies reported that the incidences of adverse effects were significantly higher in the galantamine group than in the donepezil group.^[13,16,21]^ For example, the mean incidence of nausea was 11% in the donepezil group compared to 24% in the galantamine group. Kobayashi et al reported that the incidence rates of nausea and vomiting were higher in the galantamine group than in the donepezil group but lower than in the rivastigmine group; moreover, the risk of diarrhea and dizziness was lower in the galantamine group than in the rivastigmine and donepezil group.^[26]^

Seven SR/meta-analyses reported dropout rates in patients with AD. Kurz and Van Baelen reported that the placebo group and the 16 mg/d galantamine group did not show any significant differences in the overall trial discontinuation rates (OR = 1.43, 95% CI [0.94, 2.18]) and the trial discontinuation rates because of adverse effects (OR = 0.97, 95% CI [0.51, 1.86]).^[14]^ The results of 5 studies showed that the risk of withdrawal in the AD patients was significantly higher in the galantamine group than in the placebo group; moreover, AD subjects treated with a higher dose of galantamine were more likely to discontinue treatment than those treated with lower doses of galantamine.^[17,18,25,31,32]^ Lanctôt et al compared the withdrawal rates of donepezil, rivastigmine, and galantamine and reported that the rate of withdrawal was highest in the galantamine group than the other groups (0.14, 95% CI [0.08, 0.21]).^[13]^

### 3.5. Economic evaluation of galantamine treatment in AD patients

Bond et al developed a 3-state Markov model suitable for the UK AD population and concluded in the HTA report published in 2012 that the treatment cost was lowest for galantamine but the QALY benefit was highest for donepezil.^[50]^ In another HTA report, a Markov model was used to model the AChEIs based on 2 studies that investigated the cost-effectiveness of galantamine and another study that investigated the cost-effectiveness of donepezil and carboplatin. The findings established that donepezil, galantamine, and carboplatin showed small but demonstrable clinical benefits and cost-efficacy, but there was insufficient evidence to differentiate between the cost-effectiveness of the AChEIs.^[51]^

Next, we analyzed studies that evaluated the cost-effectiveness of galantamine in the treatment of AD. We obtained 9 studies that used the AHEAD model to assess the cost-effectiveness of galantamine based on data from clinical trials, and reported that galantamine prolonged the time for patients to enter FTC.^[34–41,43]^ Cost-effectiveness was assessed using the DES model by 2 studies,^[44,46]^ Markov model by 1 study,^[47]^ cost-effectiveness analysis by 1 study,^[42]^ and cost-consequence analysis by 1 study.^[45]^

In summary, 9 economic evaluation studies of AD cohorts in different countries concluded that galantamine prolonged the time for the AD patients needing FTC.^[35,37–40,42,43,45,47]^ Therefore, galantamine treatment offset the cost of treatment, saved on full-time care costs and improved the quality of life of the patients. Based on 5 studies^[34,36,37,40,47]^ that reported QALYs and 3 studies^[37,40,47]^ that reported Incremental Cost-Effectiveness Ratios, galantamine increased the QALYs of AD patients compared to those without treatment or those who underwent placebo-controlled therapy. The HTA reports and economic studies that were included in this study were from other countries and not from local Chinese studies. Therefore, these findings can be used as reference but cannot be used to define Chinese healthcare policy.

## 4. Discussion

According to the 2021 Global Health Estimates, AD and other forms of dementia rank seventh among the top ten causes of deaths worldwide.^[52]^ In China, estimates show 15.07 million cases of dementia among subjects aged 60 and above, including 9.83 million cases of AD.^[53]^ The number of AD patients is estimated to be 20.75 million in 2030, 26.87 million in 2040, and 30.03 million in 2050.^[54]^ These rising numbers place a significant burden on healthcare systems, leading to increased demand for long-term care, specialized medical services, and support for caregivers. The increasing prevalence of AD not only strains healthcare resources but also challenges the sustainability of health insurance systems and social security networks. Moreover, it highlights the critical importance of investing in research and development for new therapeutic approaches, as well as preventive strategies to mitigate the disease’s impact. So there is an imperative to accelerate the development of innovative treatments and enhance public health initiatives like early diagnosis and screening, prevention strategies, research and development, and so on to address this growing crisis. AChEIs such as galantamine, donepezil, rivastigmine are the first-line pharmacotherapy for AD patients. Galantamine, is a highly efficient, safe, and reversible acetylcholinesterase competitive inhibitor that increases acetylcholine levels in the brain, thereby improving the function of the postsynaptic neurons and alleviates or reduces the clinical symptoms of patients.^[55]^

This rapid HTA study analyzed the efficacy, safety, and cost-effectiveness of galantamine for the treatment of AD patients. Galantamine treatment showed good therapeutic effects and significantly improved the cognitive function, daily living ability, mental behavior, and global functioning of patients with AD compared to the placebo. Heterogeneity of AD patient populations, interventions, dosage, and duration of treatment, as well as different criteria for assessing efficacy, may lead to different results in different studies. Therefore, it was not possible to infer from the currently available data whether the therapeutic efficacy of galantamine was superior or inferior to donepezil and rivastigmine. In addition, heterogeneity can make studies less comparable with each other and affect judgements about the strength of evidence from studies. When studies differ in more than 1 way, it is difficult to directly compare the results of different studies and to determine which studies provide more reliable evidence. In terms of safety, galantamine-treated AD patients showed a higher incidence of adverse gastrointestinal effects such as nausea and vomiting compared with the placebo. Compared with the other AChEIs, galantamine showed a higher incidence of adverse gastrointestinal effects than donepezil but a lower rate than rivastigmine; moreover, withdrawal rates were higher for those treated with galantamine than those treated with donepezil or rivastigmine.

The results of established pharmacoeconomic studies showed that galantamine prolonged the time for patients to enter FTC, thereby reducing the cost required for FTC and prolonging the QALYs in patients with mild to moderate AD. Galantamine also reduced the direct healthcare costs compared with the placebo. The treatment outcomes varied between countries and regions because of different health resource conditions. For example, data from Canada showed that galantamine reduced the duration of FTC by 10 percent. Besides, approximately 5.6 patients with mild to moderate disease had to be treated to avoid FTC for 1 year and resulted in an average savings of C$788 (US$528) per patient.^[35]^ In the UK, galantamine treatment reduced the average cost of FTC (over 60 months) by £1257, but these savings were not enough to compensate for an average additional £2487 in medication costs and £507 in monitoring costs.^[41]^ There is a lack of relevant pharmacoeconomic studies in mainland China. Therefore, it is possible to refer to the modeling methods used by different countries for studying the economic effects of AD-related therapeutic drugs. Moreover, a suitable economic evaluation model needs to be developed for analyzing galantamine use in China by evaluating the characteristics of the AD population in China.

Despite a systematic and comprehensive literature search, this study has some limitations. This rapid HTA study just qualitatively analyzed the included SR/meta-analysis, and economic studies, but the results may have limitations. Therefore, comprehensive HTA needs to be performed in the future, such as further studies on patient and caregiver acceptance and satisfaction with galantamine treatment, as well as analyzing its ethical and social implications. The quality rating of the included SR/meta-analyses was not high. This was due to the lack of evaluation of key entries. For example, the research plan was not registered or published prior to the systematic evaluation. This resulted in low quality literature. The included pharmacoeconomic studies were all from abroad. Therefore, the findings cannot be extrapolated directly for use in defining the Chinese healthcare policy. However, the modeling methodologies used in the literature could provide methodological references for conducting future economic evaluations targeting Chinese populations. For instance, they may inform decisions regarding model selection, the definition of health states, and the scope of included costs.

## 5. Conclusion

In summary, galantamine shows good therapeutic efficacy, cost-effectiveness, and mild adverse effects in the treatment of AD, and the treatment should be started with a small dose and gradually increased to the maintenance dose, and attention should be paid to monitoring nausea, vomiting, and other digestive system adverse reactions. With the currently available data, it is not possible to infer whether galantamine is more advantageous than donepezil, and rivastigmine. Therefore, further high-quality comparative studies are needed in the future. There is limited evidence of the economic evaluation of galantamine use in China, and further pharmacoeconomic studies are urgently needed to evaluate galantamine treatment of AD patients in China.

**Table 2:** Basic characteristics and quality evaluation of the included pharmacoeconomic studies.

Study	Country	Research perspective	Evaluation methodology	Time-frame	Decision models	Comparison group	Control group	Number of compliant items for CHEERS
Getsios et al (2001)^[34]^	Canada	Health system perspective	–	10 years	AHEAD	Galantamine	No drug therapy	22
Garfield et al (2002)^[35]^	Sweden	Medicare payer perspective	–	10 years	AHEAD	Galantamine	No drug therapy	21
Caro et al (2002)^[36]^	Netherlands	Whole-of-society perspective	–	10 years	AHEAD	Galantamine	No drug therapy	22
Ward et al (2003)^[37]^	United Kingdom	Medicare payer perspective	Cost-utility analysis	10 years	AHEAD	Galantamine	No drug therapy	22
Migliaccio-Walle et al (2003)^[38]^	America	Medicare payer perspective	–	10 years	AHEAD	Galantamine	No drug therapy	26
Caro et al (2003)^[39]^	Canada	Health system perspective	–	10 years	AHEAD	Galantamine, Donepezil, Carboplatin	No drug therapy	26
Caro et al (2004)^[40]^	Australia, Canada, Finland, New Zealand, Sweden, Netherlands and United Kingdom	Whole-of-society perspective	–	10 years	AHEAD	Galantamine	Placebo-controlled therapy	23
Green et al (2005)^[41]^	United Kingdom	Medicare payer perspective Health system perspective	Cost-utility analysis	5 years	AHEAD	Galantamine, Donepezil, Carboplatin	Placebo-controlled therapy	24
Suh et al (2008)^[42]^	South Korea	Whole-of-society perspective	Cost-effectiveness analysis	52 weeks	–	Galantamine	Placebo-controlled therapy	19
Suh et al (2009)^[43]^	South Korea	Medicare payer perspective	Cost-utility analysis	5 years	AHEAD Markov	Galantamine	placebo-controlled therapy	18
Guo et al (2010)^[44]^	German	Health system perspective	–	10 years	DES	Galantamine, non-pharmacological treatment, ginkgo biloba	Non-pharmacological treatment and ginkgo biloba	22
Wimo et al (2012)^[45]^	Sweden	Whole-of-society perspective	Cost-consequence analysis	6 months	–	Galantamine	Placebo-controlled therapy	15
Kongpakwattana and Chaiyakunapruk (2020)^[46]^	Thailand	Whole-of-society perspective	–	10 years	–	Galantamine	Placebo-controlled therapy	23
Yunusa et al (2021)^[47]^	America	Whole-of-society perspective Medicare payer perspective	Cost-utility analysis	10 years	Markov	Donepezil, Galantamine, Carboplatin, Memantine	Cross-reference	21

**Table 3:** Basic characteristics and quality evaluation of the included HTA reports.

Institution	Country	Evaluation time	Intervention measure	Control measure	Number of compliant items for HTA checklist
NICE^[48]^	United Kingdom	2001	Donepezil, galantamine or rivastigmine	Placebo or each other or non-drug	9
IQWiG^[49]^	Germany	2007	Donepezil, galantamine and rivastigmine	Placebo or direct comparisons	14
NICE^[50]^	United Kingdom	2012	Donepezil, galantamine, rivastigmine and memantine	Placebo or best supportive care or head-to-head comparisons	11
NICE^[51]^	United Kingdom	2018	Donepezil, galantamine, rivastigmine and memantine	Placebo or best supportive care	9

IQWiG = Institut fur Qualitat und Wirtschaftlichkeit im Gesundheitswesen, NICE = National Institute for Health and Care Excellence.

## Acknowledgments

We want to thank all the authors who participated in this study.

## Author contributions

**Conceptualization:** Hui Hua, Rong Jiang.

**Data curation:** Bingke Xing, Jingran Yang, Rong Jiang.

**Formal analysis:** Bingke Xing, Jingran Yang, Rong Jiang.

**Methodology:** Bingke Xing, Hui Hua, Rong Jiang.

**Writing – original draft:** Bingke Xing, Jingran Yang.

**Writing – review & editing:** Bingke Xing, Jingran Yang, Rong Jiang.

## Supplementary Material


