# Supplementary material for: Rapid health technology assessment of galantamine for the treatment of Alzheimer’s disease: A review
Source: Medicine (Baltimore). 2025 Jun 6;104(23):e42744. doi: 10.1097/MD.0000000000042744 (PMC12150971; doi:10.1097/MD.0000000000042744)
Supplement: Supplementary file 1 [file medi-104-e42744-s001.docx]

**Supplementary Table 1 Electronic search strategy**

| English Search terms | |
| --- | --- |
| 1 | Dementia [Mesh] OR Alzheimer Disease [Mesh] OR Cognition Disorders [Mesh] OR dementia*[Title/Abstract] OR Alzheimer*[Title/Abstract] OR cognitive disorder* [Title/Abstract] OR amentia* [Title/Abstract] |
| 2 | "galantamine"[MeSH Terms] OR "galantamine"[All Fields] OR "galanthamine"[All Fields] OR "galanthamine"[MeSH Terms] OR "nivalin"[All Fields] OR "reminyl"[All Fields] |
| 3 | (("systematic review" [Publication Type] OR "systematic reviews as topic" [MeSH Terms] OR "systematic review" [All Fields]) OR ("meta-analysis"[Publication Type] OR "meta-analysis as topic" [MeSH Terms] OR "meta-analysis"[All Fields]) OR ("economics" [MeSH Subheading] OR "economics" [All Fields] OR "cost" [All Fields] OR "costs and cost analysis" [MeSH Terms] OR ("cost" [All Fields] AND "analysis" [All Fields]) OR "costs and cost analysis" [All Fields]) |
| 4 | 1 AND 2 AND 3 |
