# Supplementary material for: Rapid health technology assessment of galantamine for the treatment of Alzheimer’s disease: A review
Source: Medicine (Baltimore). 2025 Jun 6;104(23):e42744. doi: 10.1097/MD.0000000000042744 (PMC12150971; doi:10.1097/MD.0000000000042744)
Supplement: Supplementary file 2 [file medi-104-e42744-s002.docx]

**Supplementary Table 2 Quality assessment results of the included SR/meta-analyses**

| **Study** | **Evaluation of items** | | | | | | | | | | | | | | | | **Quality Levels** |
| --- | --- | --- | --- | --- | --- | --- | --- | --- | --- | --- | --- | --- | --- | --- | --- | --- | --- |
|  | **1** | **2** | **3** | **4** | **5** | **6** | **7** | **8** | **9** | **10** | **11** | **12** | **13** | **14** | **15** | **16** |  |
| Krista 2003 | Yes | No | No | Partial Yes | No | Yes | Yes | Partial Yes | Partial Yes | No | Yes | No | Yes | Yes | Yes | Yes | Low |
| Kurz 2004 | Yes | No | Yes | No | No | No | No | Partial Yes | Partial Yes | No | Yes | No | No | Yes | No | No | Very low |
| Robin 2005 | Yes | No | Yes | Yes | No | No | No | Partial Yes | No | No | Yes | No | No | Yes | No | No | Very low |
| Takeda 2006 | Yes | Partial Yes | Yes | Yes | Yes | Yes | No | Yes | Yes | Yes | Yes | No | Yes | No | No | Yes | Very low |
| Loy 2006 | Yes | Partial Yes | Yes | Partial Yes | Yes | Yes | Yes | Yes | Yes | No | Yes | Yes | Yes | Yes | No | Yes | Low |
| Richard 2008 | Yes | No | Yes | Partial Yes | No | Yes | Yes | Partial Yes | Yes | No | Yes | Yes | Yes | Yes | Yes | Yes | Low |
| Noll 2008 | Yes | No | Yes | Partial Yes | No | Yes | Yes | Partial Yes | Yes | No | Yes | Yes | Yes | No | No | Yes | Very low |
| Joanne 2009 | Yes | No | Yes | Partial Yes | No | No | Yes | Yes | No | No | No meta-analysis conducted | No meta-analysis conducted | No | No | No meta-analysis conducted | Yes | Very low |
| Lockhart 2009 | Yes | No | Yes | Partial Yes | Yes | Yes | Yes | Partial Yes | Yes | No | No meta-analysis conducted | No meta-analysis conducted | Yes | Yes | No meta-analysis conducted | No | Low |
| Kavanagh 2011 | Yes | No | Yes | No | No | No | No | Partial Yes | No | No | Yes | No | Yes | No | No | Yes | Very low |
| Hu 2011 | Yes | No | Yes | Partial Yes | No | No | Yes | Partial Yes | Partial Yes | No | Yes | No | No | Yes | Yes | No | Very low |
| Ren 2014 | Yes | No | No | Partial Yes | Yes | Yes | Partial Yes | Partial Yes | Yes | No | Yes | Yes | Yes | Yes | Yes | No | Low |
| Jiang 2015 | Yes | No | Yes | Partial Yes | No | Yes | Yes | Partial Yes | Yes | No | Yes | Yes | Yes | Yes | Yes | Yes | Low |
| Hisanori 2016 | Yes | No | Yes | Partial Yes | Yes | Yes | Yes | Yes | No | No | Yes | No | No | Yes | Yes | Yes | Very low |
| Niu 2017 | Yes | No | No | Partial Yes | No | Yes | Yes | Partial Yes | Yes | No | Yes | Yes | Yes | Yes | Yes | Yes | Low |
| Andrea 2018 | Yes | Yes | Yes | Yes | Yes | Yes | Yes | Partial Yes | Yes | No | Yes | Yes | Yes | Yes | Yes | Yes | High |
| Dou 2018 | Yes | No | Yes | Partial Yes | No | Yes | Yes | Partial Yes | Yes | No | Yes | No | Yes | Yes | Yes | Yes | Low |
| Liang 2018 | Yes | No | Yes | Partial Yes | Yes | Yes | Yes | Yes | Yes | No | Yes | No | No | Yes | Yes | Yes | Very low |
| Onnita 2019 | Yes | No | Yes | Partial Yes | Yes | Yes | Yes | Yes | Yes | Yes | Yes | Yes | Yes | Yes | Yes | Yes | Low |
| Li 2019 | Yes | No | Yes | Partial Yes | Yes | Yes | Yes | Yes | No | No | Yes | No | No | No | Yes | Yes | Very low |
| Zhang 2020 | Yes | No | Yes | Partial Yes | No | Yes | Yes | Partial Yes | Yes | No | Yes | No | No | No | Yes | Yes | Very low |

Note: 1. Did the research questions and inclusion criteria for the review include the components of PICO? 2. Did the report of the review contain an explicit statement that the review methods were established prior to the conduct of the review and did the report justify any significant deviations

from the protocol? 3. Did the review authors explain their selection of the study designs for inclusion in the review? 4. Did the review authors use a comprehensive literature search strategy? 5. Did the review authors perform study selection in duplicate? 6. Did the review authors perform data extraction in duplicate? 7. Did the review authors provide a list of excluded studies and justify the exclusions? 8. Did the review authors describe the included studies in adequate detail? 9. Did the review authors use a satisfactory technique for assessing the risk of bias (RoB) individual studies that were included in the review? 10. Did the review authors report on the sources of funding for the studies included in the review? 11. If meta-analysis was performed did the review authors use appropriate methods for statistical combination of results? 12. If meta-analysis was performed, did the review authors assess the potential impact of RoB individual studies on the results of the meta-analysis or other evidence synthesis? 13. Did the review authors account for RoB in individual studies when interpreting/discussing the results of the review? 14. Did the review authors provide a satisfactory explanation for, and discussion of, any heterogeneity observed in the results of the review? 15. If they performed quantitative synthesis did the review authors carry out an adequate investigation of publication bias (small study bias) and discuss its likely impact on the results of the review? 16. Did the review authors report any potential sources of conflict of interest, including any funding they received for conducting the review?
