# Supplementary material for: Rapid health technology assessment of galantamine for the treatment of Alzheimer’s disease: A review
Source: Medicine (Baltimore). 2025 Jun 6;104(23):e42744. doi: 10.1097/MD.0000000000042744 (PMC12150971; doi:10.1097/MD.0000000000042744)
Supplement: Supplementary file 3 [file medi-104-e42744-s003.docx]

**Supplementary Table 3 Quality assessment results of the included economics studies**

| **Quality assessment entries** | | **Getsios D 2001** | **Garfield FB 2002** | **Caro JJ. 2002** | **Ward A 2003** | **Migliaccio-Walle K. 2003** | **Caro J 2003** | **Caro J 2004** | **Green C 2005** | **Suh GH 2008** | **Suh GH 2009** | **Guo S 2010** | **Wimo A 2012** | **Kongpakwattana K 2020** | **Yunusa I 2021** |
| --- | --- | --- | --- | --- | --- | --- | --- | --- | --- | --- | --- | --- | --- | --- | --- |
| **1** | | Partial yes | No | No | Partial yes | Partial yes | Partial yes | Partial yes | Partial yes | Yes | Partial yes | No | No | Yes | Partial yes |
| **2** | | Yes | Yes | Yes | Yes | Yes | Yes | Yes | Yes | No | Yes | Yes | Yes | Yes | Yes |
| **3** | | Yes | Yes | Yes | Yes | Yes | Yes | Yes | Yes | Yes | Yes | Yes | Yes | Yes | Yes |
| **Method** | **4** | Yes | Yes | Yes | Yes | Yes | Yes | Yes | Yes | Yes | Yes | Yes | Yes | Yes | Yes |
|  | **5** | Yes | Yes | Yes | Yes | Yes | Yes | Yes | Yes | Yes | No | Yes | Yes | Yes | Yes |
|  | **6** | Yes | Yes | Yes | Yes | Yes | Yes | Yes | Yes | Yes | Yes | Yes | Yes | Yes | Yes |
|  | **7** | Yes | Yes | Yes | Yes | Yes | Yes | Yes | Yes | Yes | Yes | Yes | Yes | Yes | Yes |
|  | **8** | / | / | / | / | Yes | / | / | Yes | Yes | Yes | / | / | Yes | / |
|  | **9** | Yes | Yes | Yes | Yes | Yes | Yes | Yes | Yes | Yes | Yes | Yes | Yes | Yes | Yes |
|  | **10** | Yes | Yes | Yes | Yes | Yes | Yes | Yes | Yes | No | Yes | Yes | / | Yes | Yes |
|  | **11** | Yes | Yes | Yes | Yes | Yes | Yes | Yes | Yes | Yes | Yes | Yes | Yes | Yes | Yes |
|  | **12** | Yes | Yes | Yes | Yes | Yes | Yes | Yes | Yes | Yes | Yes | Yes | Yes | Yes | Yes |
|  | **13** | Yes | Yes | Yes | Yes | Yes | Yes | Yes | Yes | Yes | Yes | Yes | Yes | Yes | Yes |
|  | **14** | Yes | Yes | Yes | Yes | Yes | Yes | Yes | Yes | Yes | Yes | Yes | Yes | Yes | Yes |
|  | **15** | Yes | Yes | Yes | Yes | Yes | Yes | Yes | Yes | No | Yes | No | No | Yes | No |
|  | **16** | Yes | Yes | Yes | Yes | Yes | Yes | Yes | Yes | Partial yes | Yes | Yes | NA | Yes | Yes |
|  | **17** | Yes | Yes | Yes | Yes | Yes | Yes | Yes | Partial yes | Partial yes | Yes | Yes | NA | Yes | Yes |
|  | **18** | Yes | NA | NA | NA | Yes | Yes | Yes | NA | NA | NA | Yes | NA | NA | NA |
|  | **19** | NA | NA | NA | NA | NA | Yes | Yes | NA | NA | NA | Yes | NA | NA | NA |
|  | **20** | Yes | Yes | Yes | NA | Yes | Yes | NA | Yes | NA | Yes | Yes | NA | Yes | Yes |
|  | **21** | NA | NA | NA | NA | NA | NA | NA | NA | NA | NA | NA | NA | NA | NA |
| **Result** | **22** | Yes | Yes | Yes | Yes | Yes | Yes | Yes | Yes | Yes | Yes | Yes | Yes | Yes | Yes |
|  | **23** | Yes | Yes | Yes | Yes | Yes | Yes | Yes | Yes | Yes | Yes | Yes | Yes | Yes | Yes |
|  | **24** | Yes | Yes | Yes | Yes | Yes | Yes | Yes | Yes | Yes | Yes | Yes | NA | NA | Yes |
|  | **25** | NA | NA | Yes | NA | NA | NA | NA | NA | NA | NA | NA | NA | NA | NA |
| **Discussion** | **26** | Yes | Yes | Yes | Yes | Yes | Yes | Yes | Yes | Yes | Yes | Yes | Yes | Yes | Yes |
| **Other relevant information** | **27** | No | Yes | Yes | Yes | Yes | Yes | Yes | Yes | No | Yes | Yes | Yes | No | No |
|  | **28** | No | No | No | No | No | Yes | No | Yes | No | Yes | No | No | Yes | Yes |

Note: 1.Title; 2.Abstract; 3.Background and objectives; 4.Health economic analysis plan; 5.Study population; 6.Setting and location; 7.Comparators; 8.Perspective; 9.Time horizon; 10.Discount rate; 11.Selection of outcomes; 12.Measurement of outcomes; 13.Valuation of outcomes; 14.Measurement and valuation of resources and costs; 15.Currency, price date, and conversion; 16.Rationale and description of model; 17.Analytics and assumptions; 18.Characterizing heterogeneity; 19.Characterizing distributional effects; 20.Characterizing uncertainty; 21.Approach to engagement with patients and others affected by the study; 22.Study parameters; 23.Summary of main results; 24.Effect of uncertainty; 25.Effect of engagement with patients and others affected by the study; 26.Study findings, limitations, generalizability, and current knowledge; 27.Source of funding; 28.Conflicts of interest; NA, not applicable; /, unreported.
