# Supplementary material for: Rapid health technology assessment of galantamine for the treatment of Alzheimer’s disease: A review
Source: Medicine (Baltimore). 2025 Jun 6;104(23):e42744. doi: 10.1097/MD.0000000000042744 (PMC12150971; doi:10.1097/MD.0000000000042744)
Supplement: Supplementary file 4 [file medi-104-e42744-s004.docx]

**Supplementary Table 4 Quality assessment results of the included HTA reports**

| **Study** | **Evaluation of items** | | | | | | | | | | | | | |
| --- | --- | --- | --- | --- | --- | --- | --- | --- | --- | --- | --- | --- | --- | --- |
|  | 1 | 2 | 3 | 4 | 5 | 6 | 7 | 8 | 9 | 10 | 11 | 12 | 13 | 14 |
| NICE, 2001 | No | Yes | No | No | Yes | Yes | Yes | Yes | Yes | No | Partial Yes | No | Yes | Yes |
| IQWiG, 2007 | Yes | Yes | Yes | Yes | Yes | Yes | Yes | Yes | Yes | Yes | Yes | Yes | Yes | Yes |
| NICE, 2012 | Yes | Yes | No | No | Yes | No | Yes | Yes | Yes | Yes | Partial Yes | Yes | Yes | Yes |
| NICE, 2018 | No | Yes | Yes | No | No | No | Yes | Yes | Yes | No | Partial Yes | Yes | Yes | Yes |

Note: 1. Are there appropriate contact details for provision of further information? 2. Are those who prepared the HTA report identified as authors or in other ways? 3. Is there a statement regarding conflict of interest? 4. Is there a statement on whether the report has been externally reviewed? 5. Is there a short summary that can be understood by the non-technical reader? 6. Is reference made to the policy question that is addressed? 7. Is reference made to the research question(s) that is/are addressed? 8. Is the scope of the assessment specified? 9. Is there a description of the health technology that has been assessed? 10. What sources of information have been used? 11. Is there information on the basis for the assessment and interpretation of selected data and information? 12. Are the findings of the assessment discussed? 13. Are the conclusions from the assessment clearly stated? 14. Are there suggestions for further action?
